# The Slovak Revised Dyadic Adjustment Scale: validation, factor structure, and population norms

**DOI:** 10.3389/fpsyg.2026.1796877

**Published:** 2026-05-14

**Authors:** Júlia Halamová, Lívia Kernová

**Affiliations:** Faculty of Social and Economic Sciences, Institute of Applied Psychology, Comenius University in Bratislava, Bratislava, Slovakia

**Keywords:** confirmatory factor analysis, couples, dyadic adjustment, factor structure, Mokken analysis, psychometric properties, RDAS, validation

## Abstract

The Revised Dyadic Adjustment Scale (RDAS) is a widely used 14-item measure of relationship adjustment. This study aimed to translate, adapt, and validate the Slovak version of the RDAS. The sample consisted of 1,090 individuals (545 couples) from Slovakia. Internal consistency was excellent for the total scale (*α* = 0.87, *ω* = 0.87) and subscales (Consensus: *α* = 0.84; Satisfaction: *α* = 0.81; Cohesion: *α* = 0.71). Confirmatory factor analysis supported the original three-factor structure (CFI = 0.925, TLI = 0.908, RMSEA = 0.072, SRMR = 0.053). Construct validity was demonstrated through strong correlations with the Couples Satisfaction Index (*r* = 0.81, *p* < 0.001). Mokken scale analysis confirmed adequate scalability (*H* = 0.375). Population norms are provided; notably, the Slovak sample mean (*M* = 48.89, SD = 9.43) suggests that United States-derived clinical cutoffs may not be directly applicable to Slovak populations, and percentile-based interpretive guidelines are recommended. Sensitivity analyses correcting for within-couple non-independence (design-effect adjusted CFA and GEE correlations) confirmed robustness of all findings. The Slovak RDAS demonstrates sound psychometric properties and is suitable for assessing dyadic adjustment in Slovak-speaking populations.

## Introduction

### Slovak translation and validation of the Revised Dyadic Adjustment Scale

Assessing relationship quality is fundamental to both clinical practice and research on romantic partnerships. The Dyadic Adjustment Scale (DAS; Spanier, 1976) has long been the gold standard for measuring marital adjustment, but concerns about item redundancy and subscale validity led to the development of the Revised Dyadic Adjustment Scale (RDAS; Busby et al., 1995). The RDAS is a brief 14-item instrument that maintains the multidimensional structure of the original DAS while demonstrating superior psychometric properties.

The RDAS measures three dimensions of dyadic adjustment: Consensus (6 items), Satisfaction (4 items), and Cohesion (4 items). Consensus assesses agreement between partners on important matters including decision-making, values, and affection. Satisfaction evaluates relationship stability and conflict frequency. Cohesion measures shared activities and quality communication. This three-factor structure has been consistently replicated across diverse samples (e.g., Anderson et al., 2014; Crane et al., 2000).

The RDAS has been validated in numerous languages and cultures, including Spanish (Herrero-Fernández, 2016), Portuguese (Hollist et al., 2012), and Persian (Holakouie-Naieni et al., 2020). These cross-cultural validations have generally supported the original factor structure and demonstrated acceptable reliability coefficients. However, no Slovak adaptation has been published to date, limiting its use with Slovak-speaking populations.

Recent cross-cultural research has demonstrated that romantic relationship dynamics, while sharing universal features, are also shaped by cultural factors including individualism–collectivism orientations, religiosity, and socioeconomic conditions (Sorokowski et al., 2023). Large-scale studies spanning multiple countries have found that modernization, collectivism, and gender equality significantly predict relationship experiences across cultures (Kowal et al., 2024). These findings underscore the importance of establishing measurement equivalence when adapting relationship instruments to new cultural contexts. Slovakia represents a Central European post-socialist country characterized by a blend of traditional family values and ongoing modernization processes. Slovak culture emphasizes family cohesion and commitment, with marriage remaining a central social institution despite increasing secularization since the 1990s (Potančoková et al., 2008). These cultural characteristics may influence how relationship quality is perceived and reported, making local validation essential for accurate assessment (cf. Xu et al., 2016).

Recent validation studies have extended the RDAS to diverse clinical and community populations. The Persian validation (Holakouie-Naieni et al., 2020) demonstrated strong psychometric properties among infertile patients, while Ethiopian adaptations (Geda and Belay, 2022) confirmed the factor structure among Oromo-speaking families. Additionally, contemporary research has highlighted the RDAS’s sensitivity to detecting relationship distress in various clinical contexts, including couples coping with chronic illness (Tompkins et al., 2013) and those receiving couple therapy (Anderson et al., 2014). The establishment of clinical cutoff scores (Crane et al., 2000) and reliable change indices (Anderson et al., 2014) has enhanced the RDAS’s utility for treatment outcome assessment.

In psychometric research, there has been growing recognition that classical test theory approaches alone may not fully capture the measurement properties of psychological instruments (Franco et al., 2022). Mokken scale analysis (MSA) has emerged as a valuable complementary method, particularly for scales with ordinal response formats (Sijtsma and van der Ark, 2017). Unlike parametric item response theory models, MSA makes fewer assumptions about the functional form of item characteristic curves, making it particularly suitable for examining whether items form coherent, homogeneous scales (Wind, 2022). Recent applications of MSA to psychological instruments have demonstrated its utility in identifying problematic items and assessing scale dimensionality (Kristófersdóttir et al., 2024).

Beyond classical test theory approaches, Mokken scale analysis (MSA) offers a nonparametric item response theory framework for evaluating scale properties (Sijtsma and van der Ark, 2017, 2021). MSA examines whether items form a unidimensional scale with ordinal measurement properties, providing complementary evidence for scale validity. To our knowledge, no previous study has applied MSA to the RDAS.

### The research aim

The present study aimed to: (a) translate and adapt the RDAS into Slovak, (b) evaluate its psychometric properties including reliability and validity, (c) examine the factor structure using confirmatory factor analysis, (d) apply Mokken scale analysis to assess scalability, and (e) establish Slovak population norms.

## Method

### Participants

The sample consisted of 1,090 individuals representing 545 romantic couples from Slovakia (529 mixed-sex, 16 same-sex). Both partners completed measures independently. Participants were recruited via an external survey agency representing different age groups and regions of Slovakia. The sample included 538 men (49.4%) and 551 women (50.6%), and 1 non-binary individual. The majority were married (78.4%; *n* = 855), while 21.6% (*n* = 235) were cohabiting. Mean relationship duration was 19.60 years (SD = 13.51, range = 1–58 years). Age distribution across five categories was: 18–25 years (9.2%), 26–35 years (27.7%), 36–45 years (25.7%), 46–55 years (19.2%), and 56 + years (18.3%). Education levels ranged from primary (0.9%) to doctoral (3.9%), with the majority having secondary (41.1%) or university (34.1%) education. Participants were distributed across all eight Slovak regions.

### Measures

#### Revised Dyadic Adjustment Scale (RDAS)

The RDAS (Busby et al., 1995) is a 14-item self-report measure of relationship adjustment. Items assess Consensus (6 items; e.g., “Making major decisions”), Satisfaction (4 items; e.g., “How often do you and your partner quarrel?”), and Cohesion (4 items; e.g., “Have a stimulating exchange of ideas”). Total scores range from 0 to 69, with higher scores indicating better adjustment. Established cutoff scores for relationship distress are: Total ≤48, Consensus ≤22, Satisfaction ≤14, and Cohesion ≤11 (Crane et al., 2000).

#### Couples Satisfaction Index (CSI-16)

The CSI-16 (Funk and Rogge, 2007) is a 16-item measure of relationship satisfaction with strong psychometric properties. It was used to assess convergent validity of the RDAS. In the current sample, the CSI-16 demonstrated excellent internal consistency (*α* = 0.95). While no formal Slovak validation exists so far, the high reliability and strong theoretical basis of the CSI-16 support its use as a convergent validity criterion.

### Procedure

The RDAS was translated into Slovak following established guidelines (Beaton et al., 2000). Two bilingual translators independently translated the original English version into Slovak. A third translator reconciled discrepancies to create a consensus version. This version was back-translated into English. The back-translations were compared with the original, and minor adjustments were made to ensure semantic equivalence. The final Slovak version was reviewed by an expert panel for content validity.

Data collection was conducted online at the end of 2023. Both members of each couple completed the questionnaires independently. The study protocol was approved by the Ethics Committee of the Faculty of Social and Economic Sciences at Comenius University Bratislava (FSEV 715/-2/2023/SD-CIII/1). All participants provided online written informed consent.

### Data analysis

Internal consistency was evaluated using Cronbach’s alpha and McDonald’s omega. Confirmatory factor analysis (CFA) was conducted using maximum likelihood estimation to test the three-factor structure. Model fit was evaluated using the Comparative Fit Index (CFI > 0.90), Tucker-Lewis Index (TLI > 0.90), Root Mean Square Error of Approximation (RMSEA < 0.08), and Standardized Root Mean Square Residual (SRMR < 0.08). Construct validity was assessed through Pearson correlations with the CSI-16. All analyses were conducted using JASP version 0.18.3 (JASP Team, 2024), R version 4.3.2 with the Mokken package (van der Ark, 2012), and Python 3.12 with statsmodels for generalized estimating equations. Because both partners in each couple completed measures, responses are non-independent. To maintain comparability with prior RDAS validation studies, primary analyses were conducted at the individual level. As a sensitivity check, we also report dyadic-corrected results. Within-couple intraclass correlation coefficients (ICCs) and design effects (DEFF = 1 + ICC; Kish, 1965) were computed for each item and scale score. For CFA, the Chi-square statistic was scaled by the average DEFF across the 14 RDAS items, and fit indices were recalculated using the effective sample size (*N*_eff_ = *N*/DEFF; Stapleton, 2006). For construct validity correlations, generalized estimating equations (GEE) with an exchangeable working correlation structure and robust sandwich standard errors were used to account for within-couple clustering (Kenny et al., 2006).

Mokken scale analysis was conducted to examine scale homogeneity and monotonicity. Loevinger’s *H* coefficient was calculated for individual items (Hi) and the total scale (*H*), with *H* ≥ 0.30 indicating a weak scale, *H* ≥ 0.40 a medium scale, and *H* ≥ 0.50 a strong scale (Mokken, 1971). Population norms were established using continuous norming methods with 95% confidence intervals.

## Results

### Descriptive statistics

Descriptive statistics for the RDAS and CSI-16 are presented in Table 1. Mean scores on the RDAS total scale (*M* = 48.89, SD = 9.43) were comparable to those reported in the original validation study (*M* = 48.0, SD = 9.0; Busby et al., 1995). Notably, the sample mean was close to the established clinical cutoff of 48 (Crane et al., 2000), suggesting that cutoff scores derived from United States samples may require adjustment for Slovak clinical settings.

**Table 1:** Descriptive statistics for RDAS and CSI-16.

Scale	*M*	SD	Range	*α*	*ω*
RDAS total	48.89	9.43	15–69	0.87	0.87
Consensus	22.16	5.46	0–30	0.84	0.84
Satisfaction	15.98	2.79	4–20	0.81	0.81
Cohesion	10.75	3.38	1–19	0.71	0.70
CSI-16	63.51	15.62	—	—	—

*N* = 1,090; RDAS, Revised Dyadic Adjustment Scale; CSI-16, Couples Satisfaction Index.

### Reliability

Internal consistency was excellent for the RDAS total scale (*α* = 0.87, *ω* = 0.87) and good for the Consensus (*α* = 0.84, *ω* = 0.84) and Satisfaction (*α* = 0.81, *ω* = 0.81) subscales. The Cohesion subscale demonstrated acceptable reliability (*α* = 0.71, *ω* = 0.70), consistent with previous studies reporting slightly lower values for this subscale (Busby et al., 1995). Reliability estimates were comparable across gender. For men (*n* = 538): total scale *α* = 0.87, Consensus *α* = 0.83, Satisfaction *α* = 0.80, Cohesion *α* = 0.71. For women (*n* = 551): total scale *α* = 0.87, Consensus *α* = 0.84, Satisfaction *α* = 0.81, Cohesion *α* = 0.71.

### Construct validity

Correlations among RDAS subscales and the CSI-16 are presented in Table 2. The RDAS total score was strongly correlated with the CSI-16 (*r* = 0.81, *p* < 0.001), providing evidence of convergent validity. All subscales showed significant positive correlations with the CSI-16, with Consensus (*r* = 0.72) and Satisfaction (*r* = 0.70) demonstrating the strongest associations. When within-couple clustering was accounted for using GEE with robust standard errors, all correlations remained strong and significant (all *p* < 0.001), though slightly attenuated: RDAS Total × CSI-16 *r* = 0.77, Consensus × CSI-16 *r* = 0.69, Satisfaction × CSI-16 *r* = 0.69, Cohesion × CSI-16 *r* = 0.46.

**Table 2:** Intercorrelations among RDAS subscales and CSI-16.

Scale	1	2	3	4	5
1. RDAS total	—				
2. Consensus	0.90	—			
3. Satisfaction	0.74	0.55	—		
4. Cohesion	0.72	0.45	0.35	—	
5. CSI-16	0.81	0.72	0.70	0.50	—

All correlations significant at *p* < 0.001. Pearson correlations; GEE-corrected correlations accounting for within-couple clustering are reported in the text.

### Confirmatory factor analysis

Within-couple ICCs indicated substantial non-independence across all 14 RDAS items (ICCs ranged from 0.41 to 0.67; average ICC = 0.51) and scale scores (RDAS Total ICC = 0.69, Consensus ICC = 0.64, Satisfaction ICC = 0.58, Cohesion ICC = 0.64). The average design effect across items was DEFF = 1.51, yielding an effective sample size of *N*_eff_ = 720 for CFA.

**Figure 1:**
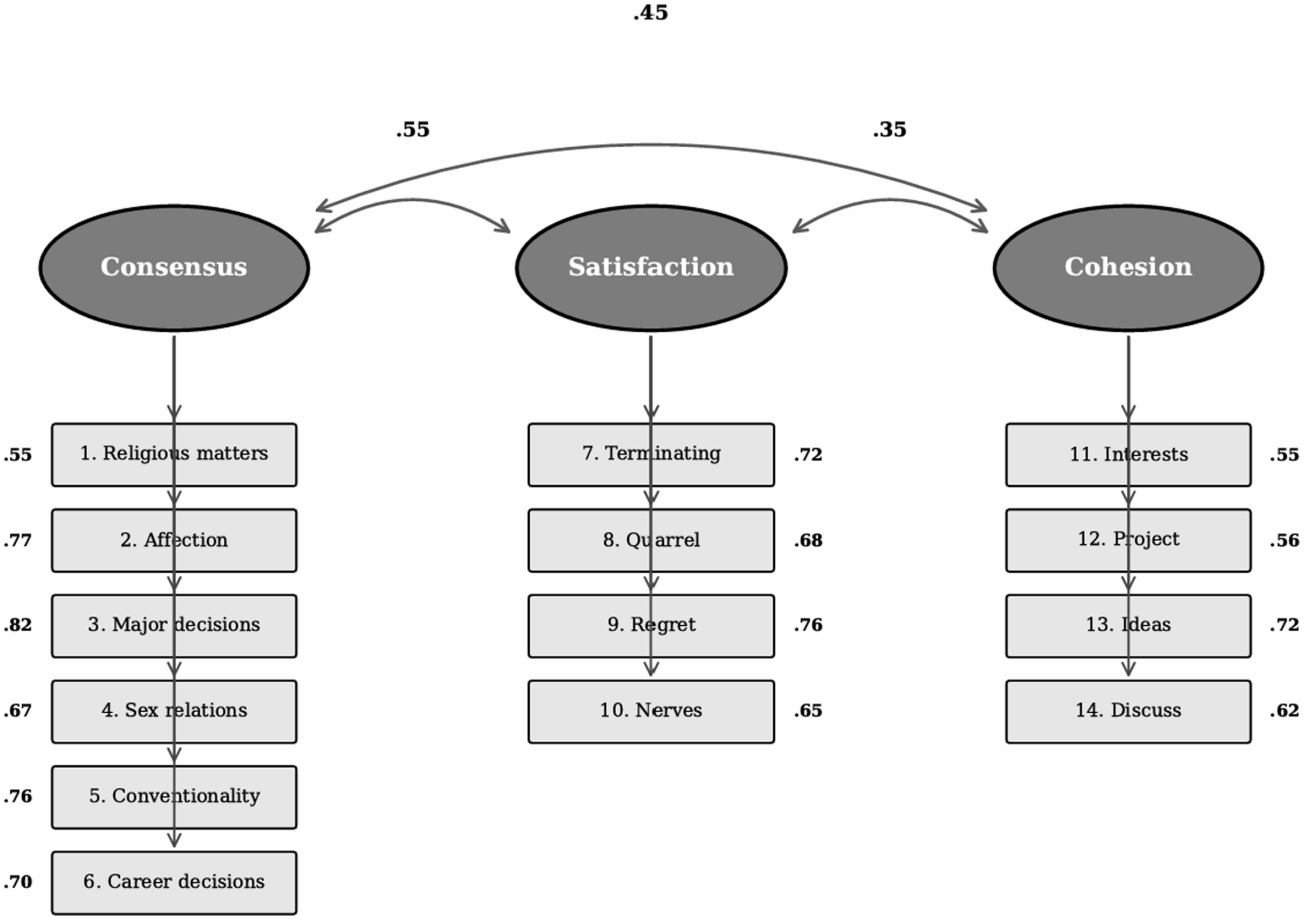
CFA three-factor model of the Slovak RDAS.

CFA results supported the original three-factor structure of the RDAS (see Figure 1). Model fit indices were acceptable: CFI = 0.925, TLI = 0.908, RMSEA = 0.072 (90% CI [0.066, 0.078]), and SRMR = 0.053. All factor loadings were significant (*p* < 0.001) and ranged from moderate to strong. When the chi-square statistic was corrected for the design effect, fit indices improved slightly: CFI = 0.931, TLI = 0.916, RMSEA = 0.069 (90% CI [0.061, 0.076]), SRMR = 0.053. All standardized factor loadings remained significant after inflating standard errors by √DEFF = 1.23 (all *p* < 0.001). Unstandardized factor loadings (with the first item of each factor constrained to 1.0 for identification) ranged from 1.00 to 1.43 for Consensus, 0.97 to 1.06 for Satisfaction, and 1.00 to 1.48 for Cohesion. Standardized factor loadings ranged from 0.55 to 0.82. Separate CFAs by gender yielded similar fit indices. Men: CFI = 0.918, TLI = 0.899, RMSEA = 0.075, SRMR = 0.056. Women: CFI = 0.930, TLI = 0.913, RMSEA = 0.070, SRMR = 0.051. These results suggest measurement consistency across gender, though formal measurement invariance testing was not conducted. The measurement model is presented in Figure 1.

### Mokken scale analysis

Mokken scale analysis was conducted to examine the scalability of the RDAS. Results are presented in Table 3. The total scale demonstrated moderate scalability (*H* = 0.375, SE = 0.013), exceeding the minimum threshold of 0.30. Item scalability coefficients (Hi) ranged from 0.208 (Item 12: Work together on a project) to 0.482 (Item 3: Making major decisions). Eleven of 14 items showed Hi values above 0.30, and most exceeded 0.35, indicating adequate homogeneity. Assumption checks were conducted following Sijtsma and van der Ark (2017). Monotonicity was evaluated using the check.monotonicity function with default minsize settings; no significant monotonicity violations were detected for any item (all #zsig = 0). Invariant item ordering (IIO) was assessed using the check.iio function. Results indicated some violations of IIO (HT coefficient = 0.31), suggesting that item ordering is not strictly invariant across trait levels, though this is common in psychological scales and does not preclude scale use. The crit statistic remained below 80 for all items, indicating no severe violations requiring item removal.

**Table 3:** Mokken scale analysis: item scalability coefficients.

Item	Hi	SE
1. Religious matters	0.299	0.020
2. Demonstrations of affection	0.462	0.016
3. Making major decisions	0.482	0.016
4. Sex relations	0.398	0.018
5. Conventionality	0.444	0.016
6. Career decisions	0.418	0.017
7. Discuss terminating relationship	0.387	0.021
8. Quarrel frequency	0.345	0.021
9. Regret marriage	0.416	0.019
10. Get on nerves	0.369	0.022
11. Outside interests	0.351	0.020
12. Work on project	0.208	0.024
13. Exchange ideas	0.287	0.020
14. Calmly discuss	0.410	0.018
Scale *H*	0.375	0.013

Hi, item scalability coefficient; SE, standard error.

Subscale analyses revealed stronger homogeneity within factors: Consensus (*H* = 0.519, SE = 0.018), Satisfaction (*H* = 0.575, SE = 0.022), and Cohesion (*H* = 0.419, SE = 0.020). All subscales exceeded the 0.40 threshold for medium scalability, with Satisfaction approaching strong scalability.

### Population norms

Slovak population norms were established using continuous norming methods. The mean RDAS total score was 48.89 (SE = 0.29, 95% CI [48.34, 49.45]) with SD = 9.43 (SE = 0.22, 95% CI [9.00, 9.85]). Stanine boundaries and percentile ranks are provided in Appendix.

The clinical cutoff of 48 established by Crane et al. (2000) was derived through discriminant analysis comparing therapy-seeking and community couples in United States samples. Our Slovak sample mean (*M* = 48.89) approximates this cutoff, which could reflect either genuine cultural differences in relationship adjustment norms or sampling characteristics. We emphasize that our normative data should not be used to establish clinical cutoffs without further validation using therapy-seeking samples and ROC analysis. The percentile-based norms provided in Appendix may assist researchers in score interpretation, but clinical screening decisions should await empirical validation with Slovak clinical populations. These observed differences may reflect cultural variations in relationship expectations between Central European and North American contexts rather than differential rates of relationship distress (cf. Körün et al., 2026). Future research should establish Slovak-specific clinical cutoffs using discriminant analysis with therapy-seeking samples.

## Discussion

This study provides the first Slovak translation and validation of the Revised Dyadic Adjustment Scale. Results support the RDAS as a reliable and valid measure for assessing dyadic adjustment in Slovak-speaking populations. The three-factor structure was confirmed through CFA, and Mokken scale analysis provided additional evidence of scale homogeneity.

Internal consistency estimates were comparable to or exceeded those reported in the original validation (Busby et al., 1995) and subsequent adaptations (e.g., Anderson et al., 2014). The slightly lower reliability of the Cohesion subscale is consistent with previous findings and may reflect the heterogeneous nature of cohesion-related behaviors (Crane et al., 2000).

Strong correlations between the RDAS total score and CSI-16 (*r* = 0.81) support convergent validity. Notably, Consensus showed a slightly stronger correlation with the CSI-16 (*r* = 0.72) than Satisfaction (*r* = 0.70), suggesting that agreement on core relationship matters—values, decision-making, and affection—may be particularly central to how Slovak couples experience relationship satisfaction. Cohesion demonstrated a moderate correlation (*r* = 0.50), indicating greater construct distinctiveness from global satisfaction. When within-couple non-independence was controlled via GEE, convergent validity correlations were slightly attenuated (e.g., RDAS Total × CSI-16 *r* = 0.77) but remained strong and significant, indicating that the pattern of results is robust to the dyadic data structure.

CFA results supported the original three-factor structure with acceptable fit indices. While the RMSEA value of 0.072 exceeded some strict criteria (<0.06), it remained within the commonly accepted range (<0.08) and is comparable to values reported in other RDAS validation studies (Herrero-Fernández, 2016), supporting the cross-cultural robustness of the RDAS conceptualization of dyadic adjustment. Importantly, when CFA fit indices were corrected for within-couple non-independence using design-effect scaling (Stapleton, 2006), all indices improved slightly (CFI = 0.931, TLI = 0.916, RMSEA = 0.069), confirming that the three-factor structure is not an artifact of inflated precision from treating coupled observations as independent.

The findings of the present study align with broader trends in cross-cultural relationship research. Recent large-scale investigations spanning multiple countries have demonstrated both universal aspects of romantic relationships and meaningful cultural variations (Sorokowski et al., 2023; Kowal et al., 2024). The Slovak RDAS validation contributes to this growing body of literature by establishing measurement properties in a Central European context.

The application of Mokken scale analysis to the RDAS represents a methodological innovation that extends beyond previous validation efforts. While most RDAS validations have relied exclusively on classical test theory and confirmatory factor analysis, the current study’s use of MSA provides complementary evidence regarding scale homogeneity and item functioning (Franco et al., 2022). The finding that subscales demonstrated stronger scalability coefficients than the total scale (Consensus *H* = 0.519, Satisfaction *H* = 0.575, Cohesion *H* = 0.419) supports their use as distinct dimensions while also validating the practice of computing a total adjustment score. This pattern of results is consistent with recent psychometric research suggesting that MSA can reveal structural properties that may be obscured by factor-analytic approaches alone (Sijtsma and van der Ark, 2021).

A novel contribution of this study is the application of Mokken scale analysis to the RDAS. The moderate overall scalability coefficient (*H* = 0.375) suggests the items form a coherent scale, though with some heterogeneity. Notably, the subscales demonstrated stronger homogeneity, supporting their use as distinct dimensions of dyadic adjustment. Items 12 and 13 (working on projects and exchanging ideas) showed lower scalability, possibly reflecting cultural differences in how couples engage in shared intellectual activities.

From a clinical perspective, the availability of a validated Slovak RDAS has important implications for relationship counseling and therapy in Slovakia. The established cutoff scores can assist clinicians in identifying couples experiencing relationship distress, while the reliable change index (Anderson et al., 2014) enables practitioners to evaluate treatment effectiveness at the individual level. Given the growing emphasis on evidence-based practice in couple therapy, having psychometrically sound assessment tools adapted to the local language and culture is essential for both screening and outcome monitoring (Crane et al., 2000). The provision of Slovak population norms further enhances the instrument’s clinical utility by allowing for normative comparisons.

The current study also has implications for research on romantic relationships in Slovakia and the broader Central European region. As relationship science increasingly recognizes the importance of cultural context in understanding relationship processes (Naeimi et al., 2024), validated measures are needed to enable meaningful cross-cultural comparisons. The Slovak RDAS can facilitate research examining how relationship quality relates to individual wellbeing, family functioning, and broader societal factors in this understudied population. Future studies may explore measurement invariance across demographic groups to further establish the generalizability of the current findings.

Several limitations should be noted. First, the sample was recruited through convenience sampling and may not be fully representative of the Slovak population. Second, although we addressed within-couple non-independence through design-effect corrections for CFA and GEE for correlations, future research could extend these analyses using multilevel CFA or Actor-Partner Interdependence Models to model partner effects more explicitly (Kenny et al., 2006). In addition, test–retest reliability was not assessed. Furthermore, the cross-sectional design precludes examination of predictive validity. Future research should address these limitations and examine measurement invariance across gender and relationship status.

## Conclusion

In conclusion, the Slovak RDAS demonstrates sound psychometric properties and is suitable for clinical and research applications. The provision of population norms facilitates interpretation of scores and identification of relationship distress. The RDAS offers clinicians and researchers a brief, validated tool for assessing dyadic adjustment in Slovak-speaking couples.

## Data Availability

The raw data supporting the conclusions of this article will be made available by the authors upon reasonable request, subject to ethical approval and applicable legal and confidentiality restrictions.

## Appendix

### Slovak population norms for the RDAS

**Table A1:** Descriptive statistics for RDAS total score norms.

Statistic	Value	SE	95% CI
*M*	48.894	0.285	[48.335, 49.454]
SD	9.425	0.215	[9.003, 9.847]

*N* = 1,090; CI, confidence interval.

**Table A2:** Stanine boundaries for RDAS total score.

Stanine	Cutoff score	SE	95% CI
1-2	32.40	0.563	[31.30, 33.50]
2-3	37.11	0.469	[36.19, 38.03]
3-4	41.83	0.384	[41.07, 42.58]
4-5	46.54	0.312	[45.93, 47.15]
5-6	51.25	0.266	[50.73, 51.77]
6-7	55.96	0.260	[55.45, 56.47]
7-8	60.68	0.295	[60.10, 61.26]
8-9	65.39	0.361	[64.68, 66.10]

*N* = 1,090; CI, confidence interval. Cutoff scores represent the upper boundary for each stanine.

**Table A3:** Percentile ranks and *Z*-scores for RDAS total score.

Raw	Percentile	SE	95% CI	*z*	SE	95% CI
15	0.05	0.05	[−0.04, 0.14]	−3.60	0.10	[−3.79, −3.40]
20	0.64	0.23	[0.19, 1.10]	−3.07	0.09	[−3.24, −2.89]
25	1.47	0.36	[0.77, 2.17]	−2.54	0.08	[−2.68, −2.39]
30	4.17	0.59	[3.02, 5.33]	−2.01	0.07	[−2.13, −1.88]
35	9.45	0.86	[7.76, 11.14]	−1.47	0.05	[−1.58, −1.37]
40	17.71	1.14	[15.47, 19.94]	−0.94	0.04	[−1.03, −0.86]
45	29.04	1.35	[26.39, 31.69]	−0.41	0.04	[−0.48, −0.34]
48	39.63	1.45	[36.79, 42.48]	−0.10	0.03	[−0.16, −0.03]
50	48.90	1.48	[46.01, 51.79]	0.12	0.03	[0.06, 0.18]
55	72.52	1.32	[69.93, 75.12]	0.65	0.03	[0.59, 0.70]
60	89.59	0.89	[87.84, 91.34]	1.18	0.03	[1.12, 1.24]
65	98.39	0.34	[97.72, 99.07]	1.71	0.04	[1.64, 1.78]
69	99.86	0.08	[99.71, 100.02]	2.13	0.05	[2.05, 2.22]

*N* = 1,090; Selected raw scores shown. Complete norms available from the corresponding author. CI, confidence interval.
